# Using Phenomenography to Tackle Key Challenges in Science Education

**DOI:** 10.3389/fpsyg.2019.01414

**Published:** 2019-06-25

**Authors:** Feifei Han, Robert A. Ellis

**Affiliations:** Office of Pro-Vice-Chancellor (Arts, Education and Law), Griffith University, Brisbane, QLD, Australia

**Keywords:** phenomenography, qualitative research method, theoretical underpinnings, second-order perspective, key challenges in science education

## Abstract

This article describes how phenomenography, as a qualitative research method, can be used to tackle key challenges in science education. It begins with an overview of the development of phenomenography. It then describes the philosophical underpinnings of phenomenographic inquiry, including ontological and epistemological roots, and its unique second-order perspective. From theoretical background to practicality, the paper uses rich examples to describe in detail the procedures of conducting a phenomenographic study, including sampling and data collection, analyzing phenomenographic data, and communicating key findings. The paper concludes by showing how the phenomenographic method can be used to develop students’ conceptual understanding of scientific concepts, to inform effective instructional design in science teaching, and to identify and improve evidence-based factors in student learning to enhance learning outcomes in science.

## Introduction

How to assist students in achieving better quality of learning in science subjects is an ongoing agenda in science education. With a purpose to impact on real-world educational practice in science education, researchers from different methodological camps bring their own ontological (why things exist the way they do) and epistemological (how learning occurs) perspectives to the advancement of theories in science teaching. However, no matter what background they come from, there are some common challenges faced by science educators today. This article draws on national reports into challenges for science education and describes a research method for addressing them known as phenomenography.

Current educational dilemmas facing science education are highlighted in national reports in the United Kingdom (Hoyle, 2016), United States (National Research Council, 2012), and in many other countries (Alberts, 2013). One of the grand challenges for science education is to improve students’ conceptual development of scientific concepts, including helping students modify their prior mistaken concepts, and/or moving novice concepts toward professional ones (Osborne et al., 2016). To achieve this goal, it is important to begin with identifying what concepts students already have, whether the concepts are aligned with scientific explanations, and if not, what aspect(s) make it variant from what is commonly understood (National Research Council, 2012). The phenomenographic method is illuminating, because the content-rich phenomenographic data can be used evaluate students’ initial understanding and the evolvement of that understanding of scientific concepts (Minasian-Batmanian et al., 2006).

Moreover, to facilitate students’ understanding of scientific concepts and guide them away from pathways that lead to misunderstandings, especially for abstract and difficult concepts, science educators should develop innovative instructional strategies from various angles in order to help students understand scientific concepts more holistically (National Research Council, 2012). Phenomenography is useful to achieve this aim because it serves as a basis for using the variation theory of learning to improve pedagogical design for presenting scientific concepts (Lo and Chik, 2016; Pang and Ki, 2016).

Another key challenge faced by science educators is to identify key aspects of student learning experience which are able to explain learning outcomes so as to take targeted actions to improve the learning experience. Using the phenomenographic method, researchers in science education have identified variations in conceptions of and approaches to learning science subjects, and perceptions of the teaching quality and learning environment, all of which account for qualitatively different learning outcomes (Hardy et al., 2014; Kapucu, 2014). Once these variations have been identified, educators can implement corresponding strategies to change the less desirable variation(s) of these elements (e.g., fragmented conceptions, surface approaches, and negative perceptions) to the more desirable ones (e.g., coherent conceptions, deep approaches, and positive perceptions) to enhance quality of science learning.

Before we unpack how to apply phenomenography in tackling these issues, we first introduce the philosophical background of the method and explains practical issues in conducting phenomenographic studies using representative examples in published studies. The following provides a brief historical account of phenomenography and how and where it has been used. It highlights theoretical underpinnings of the method and explains key procedures of conducting a phenomenographic study, including data collection, sampling methods, principles and procedures of phenomenographic analysis, and ways of communicating findings. The last section discusses how the research method can be meaningfully used to tackle the three key challenges in science education.

## Research Foci and How Phenomenography Has Been Used

Phenomenography was initially developed by a body of educational researchers in Sweden in the late 1970s to study variations of how students learn and understand concepts (Marton and Säljö, 1976a, 1976b; Marton and Svensson, 1979; Säljö, 1979). In its subsequent development, the research foci have been expanded. The method examines “qualitatively different ways in which people experience, conceptualize, perceive, and understand various aspects of, and various phenomena in the world around them” (Marton, 1986, p. 31).

Phenomenography is now known as a well-established qualitative research method and has been widely adopted to research education in multiple disciplines, such as technology (Englund et al., 2017; Hsieh and Tsai, 2017), engineering (Case and Light, 2011; Magana et al., 2012), mathematics (Kapucu, 2014; Gordon and Nicholas, 2015); and terrains beyond education, like management, computer programming, organizational studies, library and information research, nursing, medical and health care research (Yates et al., 2012; Stenfors-Hayes et al., 2013; Teeter and Sandberg, 2016). In the last couple of decades, the method has been especially appealing to science educators (Brown et al., 2006; Olympiou and Zacharia, 2012; Lee et al., 2013; Chiu et al., 2016; Howitt and Wilson, 2018).

## Theoretical Underpinnings of Phenomenography

Ontologically speaking, phenomenography believes that “an individual cannot experience without something being experienced” (Marton and Pang, 2008, p. 535). This means that phenomenographic researchers do not treat a phenomenon separately from people who experience it (Sin, 2010). Marton (2000, p. 105) further elaborated the ontology of phenomenography:

“There are not two worlds: a real, object world, on the one hand, and a subjective world of mental representations, on the other. There is only one world, a really existing world, which is experienced and understood in different ways by human beings. It is simultaneously objective and subjective.”

Using an example of approaches to learning as a research object to illustrate, phenomenographic researchers consider that the approaches adopted by students are not an inherent trait, but may vary from one learning context to another, depending on factors, such as students’ understanding of the disciplinary contents, their perceptions of the course design, and their views of the learning environment. This means that the same student may adopt a deep approach (e.g., being proactive, taking initiatives, and seeking in-depth meaning of the subject matter, Prosser and Trigwell, 1999; Vermunt and Donche, 2017) to learning *biology*, but he/she may adopt a surface approach (e.g., following formulas, rote memorization, reproducing the contents in the textbooks, and completing the learning tasks with little reflections, Prosser and Trigwell, 1999; Vermunt and Donche, 2017) to studying *chemistry*, because the student may find difficult to understand the learning goals in chemistry.

Turning toward the epistemological stance, which reflects a person’s view on the nature of knowledge, phenomenography is grounded in the “intentionality” of human behaviors, which is characterized by purposefulness and consciousness, involving different foci of an awareness of a phenomenon. Such intentionality can generate two sources responsible for the qualitative variations in an experience. For one thing, people may experience different parts of a phenomenon. For another, even if they experience the same parts, these parts may not in the foreground of their awareness (Yates et al., 2012). This is why some people can share the same experience but come away with different meanings from it.

**FIGURE 1:**
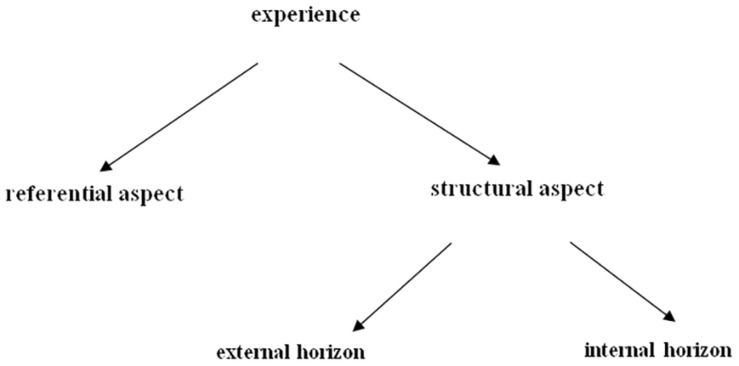
The anatomy of experience (adapted from Marton and Booth, 1997, p. 88).

The phenomenographic method present sources of variations in an unique analytical framework known as “the anatomy of experience,” which describes the two components of the conscious awareness of an experience, namely a referential aspect and a structural aspect. While the former refers to the meaning of an experience, the latter is related to the structure of that experience (Marton and Pong, 2005). The two aspects simultaneously occur and are intertwined (Marton and Booth, 1997). The structural aspect can be further distinguished between an external and an internal horizon. The external horizon, the “discernment of the whole from the context,” enables the experience to be differentiated from its context and background (Marton and Booth, 1997, p. 87); whereas the internal horizon, the “discernment of the parts and their relationships within the whole,” denotes the internal relationship of various parts in an experience, how the parts are distinctive from each other, and how the parts jointly form a cohesive entity (Marton and Booth, 1997, p. 87) (see Figure 1 for an visual representation “the anatomy of experience”). To be cognizant of all aspects of a phenomenon is to be consciously aware of its referential and structural components.

**FIGURE 2:**
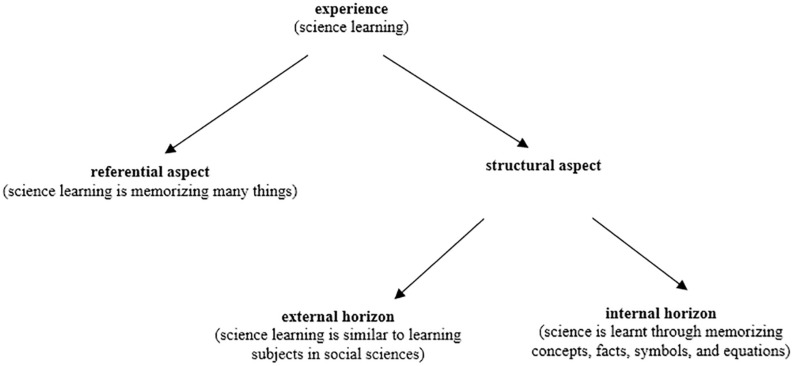
The anatomy of experience of science learning.

We use ‘conceptions of learning science’ as a research object to illustrate different aspects of “the anatomy of experience.” A student describes his/her conceptions of learning science: “When learning science, I need to memorize many concepts, facts, symbols, and equations. Sometimes, I feel that I am learning social studies such as history and language while learning science…” (Tsai, 2004, p. 1739). The learner assigns “memorizing many things” as the meaning of learning science, which is the referential aspect. The learner distinguishes “learning science” from the background of learning other subjects (i.e., external horizon of the structural aspect), even though his/her experience finds learning these subjects share similarities. The learner describes that the experience of memorizing includes a number of parts, such as concepts, facts, symbols, and equations; and recognizes that these parts together constitute the things needs to be memorized (i.e., the internal horizon of the structural aspect) in order for learning to occur. These aspects can be visually represented in the anatomy of experience of “learning science” in Figure 2.

Another important theoretical underpinning of phenomenography is its unique second-order perspective, which emphasizes the collective meaning and variations in a phenomenon *as experienced by people* (Marton and Pang, 2008). This contrasts sharply with the first-order perspective, which focuses on explicating the general and invariant essence of a phenomenon *through people* (Richardson, 1999; Marton and Pang, 2008). The detailed explanations of the first- and second-order perspectives are given by Åkerlind (2018) in the following:

“From a second-order perspective, human experience and variation in experience is the core of the investigation; from a first-order perspective, human experience is but the medium for collecting data, and variation in human experience (within the same experimental conditions) is white noise, to be filtered by statistical tests of significance to better determine the reality underlying the noise.” (p. 6)

Such fundamental difference between the first- and second-order perspectives is also reflected in the research questions addressed by phenomenography and methods adopting first-order perspective. For instance, “What are the different approaches college students adopt to learn physics?” is more suitable to be answered using the phenomenographic method, because the research purpose is to gain an understanding of *various ways* of learning physics *in the lenses of college students*. On the other hand, the research question “How do college students learn physics?” is more appropriate to be investigated using the first-order perspective, as the focus is on describing *the common features* which characterize tertiary physics learning.

Having described the theoretical underpinnings of phenomenography, the next section explains practical issues of conducting a phenomenographic study by using accessible examples.

## Key Procedures of Conducting a Phenomenographic Study

Three key procedures for conducting a phenomenographic study are described in the following: (1) data collection and sampling, (2) principles of phenomenographic data analysis, and (3) effective communication of the phenomenographic results.

### Data Collection and Sampling Methods

There are multiple ways to collect phenomenographic data, such as using semi-structured interviews, open-ended questionnaires, think-aloud methods, and observation, each of which offers different strengths and limitations to the research process. When there are a relatively large number of participants, using an open-ended questionnaire is advantageous as it is easy to administer and allows a wider range of experiences of a phenomenon to be captured. Think-aloud methods, which require participants to verbalize their thoughts while performing a task, are more suitable to uncover a process-oriented phenomenon, like carrying out a scientific experiment. While think-aloud methods are able to reflect detailed concurrent thinking, an obvious drawback is that data collection is time-consuming and the essential training of participants adds an extra burden. Used to a much lesser extent, observation is used to reflect how people perceive a phenomenon through what they act upon (Dall’Alba, 1994; Marton, 2015). Observation has a merit to collect the information of both the process (e.g., video clips and field notes of dissecting specimens in a laboratory) and the product of an activity (e.g., the dissected organs), providing triangulation from multiple data sources (Lam, 2017).

The most popular phenomenographic data collection method is semi-structured interviews, which are often conducted using a set of pre-defined interview questions as well as the information emerging from participants’ responses (Stenfors-Hayes et al., 2013). While other qualitative interviews either focus on the participant or the phenomenon itself, the phenomenographic interviews emphasize the relation between the participant and the phenomenon (Bruce, 1997). Hence, the interview questions should be carefully constructed to allow participants to reflect on their experience (Yates et al., 2012). For instance, to find out conceptions of “learning science,” a question like: “What do you understand by ‘learning science’?” is more appropriate than “What is ‘learning science’?”, because the former is on the interplay between the interviewee and science learning, whereas the latter is on science learning itself, which does not necessarily involve the interviewee’s personal experience.

To secure a rich understanding of the students’ perspectives in interviews, researchers should give them freedom to expand their understandings, and researchers should ask follow-up questions to explore interesting themes from the responses. When constructing follow-up questions, neither should researchers ask leading questions nor should they introduce ideas that has not been expressed by the interviewees to avoid collecting biased data (Åkerlind et al., 2005). A question like: “What are the differences between learning science and learning social sciences subjects?” would be leading because it presumes that learning science differs from learning social sciences subjects. A more appropriate question would be: “Do you consider learning science and learning social sciences to be the same thing? Why or why not?” However, if the interviewee has responded: “To me, science learning is quite different from learning social sciences, such as history and language,” then asking “What are the differences?” is not leading. In this scenario, researchers should explore the differences between “learning science” and “learning history” or “learning language” experienced by the interviewee rather than introducing another social sciences subject.

With regard to sampling method, phenomenographic inquiry adopts purposeful sampling, which resembles most of other qualitative methods (Marton, 1986; Booth, 1997). To select participants, researchers should consider whether the potential participants have experienced the phenomenon under investigation; and whether the number of the participants are sufficiently large for variations to be revealed. However, purposeful sampling by no means just targets a particular type of individuals, as this will result in danger of undermining variations and violating the validity of the study (Ashworth and Lucas, 2000). For instance, when a researcher intends to explore first year undergraduates’ approaches to learning science, he/she should not only target those with good academic performance in science subjects. Otherwise, opportunities to capture approaches to learning science from students with poor academic performance will be lost.

In phenomenographic research practice, using both semi-structured interviews and open-ended questionnaires to collect data is often favored as such combination allows both breadth and depth of variations to be covered in the data. Because the semi-structured interviews are able to provide rich and in-depth descriptions, whereas the open-ended questionnaires are suitable for collecting data from relatively large number of participants to cover a wider range of experience for variations to be revealed (see Kapucu, 2014; Chiu et al., 2016 as examples).

### Principles and Processes of Analyzing Phenomenographic Data

The main aim of phenomenographic data analysis is to identify a set of qualitatively different categories representing variations of individuals’ experience of a phenomenon. There are a set of special principles to follow in phenomenographic data analysis to achieve this. The most important principle is that data analysis is iterative rather than sequential (Yates et al., 2012). This principle alerts researchers to not to make quick decisions on the number of categories arising from the data. Another principle is that analyses should focus on searching for collective meaning of responses rather than describing each individual’s response (Åkerlind, 2005). Thirdly, researchers should avoid merely presenting participants’ responses without identifying variations and relations amongst them (Bruce, 1997). Interestingly, there is no singular agreed upon analytical procedure about how to analyze phenomenographic data (Ashworth and Lucas, 2000). For this reason, Table 1 summarizes the main stages proposed by different researchers. Although the number and the name of the stages vary, there are some similarities in terms of key stages.

**TABLE 1:** Phenomenographic data analysis processes.

Marton et al. (1992)	Dahlgren and Fallsberg (1991), McCosker et al. (2004)	Säljö (1997)
	**1. Familiarization:** The data are viewed for researchers to be familiar with the details of the data.	**1. Familiarization:** Similar to stage 1 in Dahlgren and Fallsberg (1991), McCosker et al. (2004)
**1. Identification:** Data which is related to the phenomenon being described are identified.	**2. Condensation:** The most representative statements are selected to uncover the patterns of the data.	**3. Identification:** Similar to stage 1 in Marton et al. (1992)
**2. Sorting:** The identified data are sorted into ‘pools of meaning’ according to similarities.	**3. Comparison:** Unpack similarities and differences to identify sources of variations.	**4. Sorting:** Similar to stage 2 in Marton et al. (1992)
**3. Contrasting and categorizing:** The ‘pools of meaning’ are contrasted, and categories are generated with descriptions.	**4. Grouping:** The statements are sorted by similarities.	
**5. Articulating:** The essence of similarities are extracted, categorized, and described.		
**6. Labeling:** The categories are represented linguistically.		
**7. Contrasting:** The categories are contrasted.	**5. Contrasting and categorizing:** Similar to stage 3 in Marton et al. (1992)	
**4. Reliability checking:** The reliability is checked by having a portion of the data coded by independent researchers and the inter-coded reliability is calculated.		**6. Reliability checking:** Similar to stage 4 in Marton et al. (1992)

Table 1 reveals that researchers seem to agree that phenomenographic data analysis commences with a stage of familiarization, which is normally realized by viewing and reading through the transcripts of the interviews or the responses in the open-ended questionnaires. The purpose of familiarization is for researchers to develop a good sense of the breadth and depth of the participants’ responses. Following familiarization stage is data reduction and condensation stage, which is given different names by different researchers [e.g., “identification” in Marton et al. (1992) and Säljö (1997); “condensation” in Dahlgren and Fallsberg (1991) and McCosker et al. (2004)]. Reduction and condensation is achieved through identifying the most relevant and important parts in the responses, allowing patterns of the responses to be revealed more easily. The third main stage is classification of responses, which is achieved through comparing and contrasting similarities and differences in order to generate an initial set of the categories. Each category should stand distinctly to reflect *the variation* of the experience rather than singular experience (Bowden, 2000). The next stage is labeling categories using appropriate descriptors which best represent the theme of each category. Due to the iterative nature of phenomenographic data analysis, classifying and labeling stages often take place multiple times, during which the initially formed categories and their descriptions are refined and modified to reach a final set of categories, which should best represent the qualitative variations of the phenomenon from the participants’ responses.

When deriving categories, it is important to remember three points in order to provide the most meaningful and transferable outcomes. First, each category should reveal some distinctness from other categories. The distinctness can be either from the referential aspect focusing on differences in the meaning or from the structural aspect focusing on different parts or combinations of parts (Marton and Booth, 1997). Second, the number of categories should be parsimonious. Third, the type of the logical relations amongst the categories should be clearly specified (Marton and Booth, 1997). The process of specifying logical relations amongst the categories helps pinpoint whether the variation is caused by: (1) failure to distinguish the phenomenon from its context; (2) unawareness of some parts of the phenomenon; (3) having different perceptions of the structural relations between the parts; or (4) a combination of these.

In Table 1, it should be noted that only Marton et al.’s (1992) and Säljö’s (1997) procedure has a stage of reliability checking. Unlike the quantitative research methods whose reliability is on replicability in other research contexts, the term of “reliability” in phenomenographic research places emphasis on consistency of assigning data using the generated categories by other researchers (Marton, 1988). Marton et al. (1992) advises that two or more researchers should apply the categories and analyze the data independently. Disagreement can be discussed and resolved to minimize researcher bias (Tight, 2016). The inter-judge reliability (also called as inter-judge communicability) can be computed based on the disagreement after the discussion (Cope, 2004).

To illustrate the key stages in phenomenographic data analysis, we use students’ responses about “conceptions of learning science” as an example (adapted from Tsai, 2004).

Extract A: *“I just have an impression that in science classes, the teachers often state manyspecial terms and formula in which I am supposed* ***to memorize****.”*

Extract B: *“The major purpose of learning science is* ***to pass the exams*** *and have high exam scores, and then get into good colleges.”*

Extract C: *“Learning science indicates the* ***acquisition of*** *scientific* ***knowledge***. *I have more knowledge derived from science instruction.”*

Extract D: *“Learning science is* ***preparing for tests***. *Science, for us, is a major subject for the College Entrance Examination.”*

Extract E: *“The purpose of learning science is* ***to acquire*** *more* ***knowledge*** *about natural phenomena and living things.”*

Extract F: *“Learning science is to acquire some knowledge and skills to solve real-life problems. Science needs* ***to be applied to*** *solve practical problems.”*

Extract G: *“When learning science, I need* ***to memorize*** *many concepts, facts, symbols, and equations. Sometimes, I feel that I am learning social studies such as history and language while learning science…”*

Extract H: *“Learning science helps us obtain knowledge. The knowledge can* ***be applied to*** *invent more products to improve the quality of our life.”*

Using the stages in Table 1 as a guide, in the familiarization and identification stages, researchers may mark or take notes of the key words (bolded in extracts A to H), such as: “to memorize” (in A), “to pass the exams” (in B), “acquisition of…knowledge” (in C), which reveal some distinct features of the conceptions. In the next stage, by comparing and contrasting these features, the responses sharing similar features, such as A and G (memorizing), B and D (preparing for tests), C and E (acquiring knowledge), and F and H (applying) can be grouped to form an initial set of categories. Then researchers can start to describe each category by paying attention to the marked key words in the responses. For instance, in the category made up by A and G, “to memorize” appears to be the main theme, which conceives learning science as memorizing different things, including “special terms,” “formula,” “concepts,” “facts,” “symbols,” and “equations.” Therefore, possible labels for this category could be “learning science is to memorize,” “learning science is a process of memorization,” or “learning science involves memorizing many things.”

### Communicating Results in Phenomenographic Research

The phenomenographic results are presented as an outcome space, which is defined as a “logically structured complex” (Marton, 2000, p. 105), “a diagrammatic representation” (Bruce, 1997, p. 87), and “a map of a territory” (Säljö, 1988, p. 44). The outcome space has two essential elements: descriptions of each category and selections of illustrative statements accompanying each category (Marton, 1994; Bowden, 2000). The outcome space can be represented in various formats, such as in tables, in diagrams, or in figures (Yates et al., 2012). Corresponding to the structural relationship between the categories, three types of outcome space are recommended in phenomenographic data presentation. The most common type is a hierarchically inclusive outcome space, in which the categories are arranged from lower-order to higher-order categories, and the lowest level represents the most simplistic way, whereas the highest level indicates the most sophisticated and developed way of experiencing the phenomenon (Tight, 2016). The outcome space can also be arranged chronologically (temporal ordering), which denotes the evolution of the participants’ experience of a phenomenon (Englund et al., 2017). The outcome space presented in a climatic order is adopted when the categories are arranged according to the level of the explanatory power (Laurillard, 1993).

In the following, we present a sample outcome space of “conceptions of learning science” (adapted from Tsai, 2004) (Table 2) and discuss the structural and referential aspects of the categories (Table 3).

**TABLE 2:** An outcome space of conceptions of learning science.

**Categories**	**Descriptions**	**Representative statements**
1. Memorizing	Memorizing definitions, formulae, laws, and special terms	*When learning science, I need to memorize many concepts, facts, symbols, and equations. Sometimes, I feel that I am learning social studies such as history and language while learning science. There are often a lot to be remembered. I often need to rehearse these concepts and equations again and again to keep them strictly in my mind.*
2. Testing	Preparing for tests, passing the examinations or achieving high scores	*The major purpose of learning science is to pass the exams and have high exam scores, and then get into good colleges.*
3. Calculating	Calculating, practicing tutorial problems, and manipulating formulae and numbers	*Learning science involves the application or rearrangement of certain formulae to compute a right answer.*
4. Increase	Knowledge acquisition and accumulation of scientific knowledge	*Learning science indicates the acquisition of scientific knowledge. I have more knowledge derived from science instruction.*
5. Applying	Applying scientific knowledge in practical situations	*Learning science is to acquire some knowledge and skills to solve real-life problems. Science needs to be applied to solve practical problems.*
6. Understanding	Obtaining deep understanding, and constructing integrated and theoretically consistent knowledge	*Learning science needs a deep understanding of scientific knowledge. If you do not really understand, you will encounter a lot of conflict. And, you will not make sense of its concepts.*
7. Seeing in a new way	A process to get a new perspective and a new way to interpret natural phenomena	*Learning science brings new ways to see natural phenomena for me. Often, the scientific knowledge challenges my intuitions, and I finally know that I was incorrect in seeing something.*

**TABLE 3:** Structural relations amongst categories of conceptions of learning science.

**Categories**	**Forms of knowledge acquisition**		**Motivational orientation**		**Evaluation of learning outcomes**	
	**Reproducing**	**Extending and developing**	**External**	**Internal**	**How much is learnt**	**How well it is learnt**
1. Memorizing	x		x		x	
2. Testing	x		x		x	
3. Calculating	x		x		x	
4. Increase	x			x	x	
5. Applying		x		x		x
6. Understanding		x		x		x
7. Seeing in a new way		x		x		x

As shown in Table 2, there are seven qualitatively different ways of learning science conceived by high school students. Structurally, these categories are hierarchically related, with “memorizing” as the most simplistic conception and “seeing in a new way” as the most sophisticated one in the hierarchy. The level of sophistication increases as the categories move from 1 to 7. Referentially, the categories offer qualitatively different meaning in three dimensions. In terms of forms of knowledge acquisition and standards for evaluation of outcomes, there is a marked shift between categories 1–4 and categories 5–7. While categories 1–4 consider the value for learning science is knowledge reproducing and use the quantity to evaluate learning outcomes; categories 5–7 conceive learning science as applying theories to solve real life problems and providing new perspectives to understand the nature, and these categories are more concerned with the quality of learning.

## Using the Phenomenographic Method to Tackle Challenges in Science Education

As outlined in the introduction that the phenomenographic method are suitable to tackle some current international challenges in science education, this section will explain these in detail. First, the phenomenographic method is a good way to evaluate students’ understanding of scientific concepts and identify sources of misunderstanding because the phenomenographic data not only offer rich and contextual descriptions of students’ understanding but are able to unpack a holistic understanding into “different patterns of awareness and non-awareness of component parts” (Åkerlind, 2018, p. 3), which allows the sources of misconceptions to be revealed more easily (Newton and Martin, 2013; Svensson, 2016). Educators can ask students to talk about a scientific concept and audio-record the answers, or they can ask students to write down their understanding. Then the educators can pinpoint the source of misunderstanding following the procedure we have described in “analyzing phenomenographic data.” Once these sources are found, teachers may group students according to categories of misunderstandings, and present different information to different groups of students by highlighting the parts which they are unaware of or directly explain the structural relations between the parts, depending on the sources of misunderstandings. Using an example from Lo and Chik (2016) to illustrate, in assessing students’ understanding of an astronomical occurrence – solar eclipses, students were asked if it is possible that solar eclipses occurred 12 times in a year. Student A responded that she thought that it is possible because the Moon travels around the Earth once a month, that is 12 times in a year, therefore, the Moon should block the Sun from the Earth 12 times in a year, producing 12 solar eclipses. This response reveals that the source of misunderstanding of the solar eclipses formation is her unawareness of the critical feature that “the Moon has an orbit that is tilted at an angle to the plane of the Earth’s orbit” (p. 301). Once this is identified, the teacher can highlight this critical feature that the orbit of the Moon is tilted to the Earth’s orbit in the instruction or in the learning activities. This will clarify students’ misconceptions that when the Moon is in between the Earth and the Sun, they are always on the same straight line.

Second, the phenomenographic method can be applied in science teaching to create facilitative conditions for learning difficult and abstract scientific concepts. The application of the phenomenographic method in instructional design is known as the variation theory of learning (Pang and Marton, 2013). It recognizes the qualitative variations of people’s experience and interpretation of phenomena. In applying variation theory to instruction, the general principle is to introduce the variation of a critical aspect(s) of an object of learning (e.g., a scientific concept) to enable learners to discern and focus on this aspect while keeping the other aspects (the unfocused aspects) invariant (Pang and Ki, 2016). In this process, the phenomenographic data analysis can be used to identify “the critical features and aspects, relevance structure, and patterns of variation” for the object of learning (Lo and Chik, 2016, p. 296). Phenomenographic research has identified four patterns of variation: namely *separation*, *contrast*, *generalization*, and *fusion* (Marton, 2015). Using these patterns, science teachers can manipulate the conditions of how information is presented to students in different ways to draw students’ attention to the critical aspect(s) that the students need to discern in order to learn a scientific concept. The instructors can separate the critical aspects and the non-critical aspects of a concept (*separation*); keep some critical aspects of a concept invariant (*generalization*) while another varies (*contrast*); clarify the interrelationships amongst the critical aspects and the part-whole relationships within a concept (*fusion in the internal horizon*); and delineate the relationship between a concept and its background (*fusion in the external horizon*) (Lo and Chik, 2016). Pang and Ling (2012), for example, described an instructional design, which aimed to help secondary school students understand an important chemical concept – “whether the volume of the reactant or the concentration level of the reactant affects the rate of a chemical reaction.” It showcased how chemistry teachers kept some critical aspects invariant while another varied in two sets of experiments. In the first set, the mass of CaCO_3_ was kept invariant, the concentration of acid was kept invariant, but the volume of acid varied. These experiments helped students discern that “the volume of the reactant does not affect the rate of the chemical reaction when the concentration level of the reactant remains the same.” In the second set of experiments, the mass of CaCO_3_ was kept invariant, the volume of acid was kept invariant, but the concentration of acid varied. The second set of experiments enabled students to discern that “the concentration level of the reactant affects the rate of the chemical reaction even though the volume of the reactant remains the same.”

Third, to improve the quality of science learning, another issue for science educators to deal with is continuous identification of factors (e.g., students’ approaches to learning science, and how students perceive science learning environment) which contribute to the learning outcomes (Hardy et al., 2014). Past research in science education has consistently demonstrated that qualitatively different conceptions of learning science are logically related to how students go about learning it, and levels of learning outcomes (Minasian-Batmanian et al., 2006). These studies reported that students who hold fragmented conceptions of learning science tend to adopt more surface approaches, and achieve relatively poorly; whereas those with cohesive conceptions are more likely to adopt deep approaches to learning science, and have relatively better academic performance. The phenomenographic method can be used to identify variations of other factors in students’ learning experience, such as students’ perceptions of the course design, students’ understanding of laboratory experiments, and students’ approaches to teamwork and collaborations. This may help science educators decide which factor(s) they should act upon to move students from less undesirable to more desirable variation of learning experience in order to enhance their learning outcomes in science subjects.

For example, once teachers find that some students believe that learning science does not have any practical applications in everyday life at all, and that science learning is merely rote memorization of scientific formula without needing to understand the principles behind them, teachers may try to help students change such fragmented conceptions and relate science learning to solving real life issues. Teachers may design learning activities for students to conduct scientific investigation of practical problems in their lives and local communities related to a class theme, such as “Does the weather affect your pulse?”, “Which soil is the best growing medium?”, “Does exercise improve your memory?” (Forbes and McCloughan, 2010; Forbes and Skamp, 2019). Through participation in authentic scientific activities, students will become more engaged in every process of scientific inquiry, including observing phenomena related to personal and societal contexts, questioning, predicting, testing, collecting, analyzing, reasoning and arguing, so that they may start to value scientific investigation in finding real life solutions and appreciate the beauty of scientific reasoning. It is through the phenomenographic method, which is concerned with the interplay between a phenomenon and people who experience it, science educators are able to continuously locate and modify undesirable variations of learning experience to help students learn science better.

## Conclusion

The purpose of this article has been to introduce readers to the phenomenographic research method, which can be usefully designed to tackle contemporary challenges in science education. A fundamental purpose of the method is to describe people’s collective experience of the world and variations in that collective experience. This is particularly useful for educators interested in understanding why some students learn more deeply and successfully than others, even though they all experience the same course assessment and activities. In order to provide science educators a theoretical appreciation of the method and capacity to implement it in practice, we have described the origin and development of the phenomenographic framework, including its ontological and epistemological assumptions, and its unique second-order perspective. We have then illustrated the key procedures of conducting phenomenographic research using examples. The article continues with an account of how the method can be applied to: (1) identify sources of students’ misunderstanding of scientific concepts; (2) implement effective instructional design for teaching difficult and abstract scientific concepts; and (3) locate actionable elements in student experience of learning science which are likely to impact on quality of learning outcomes. The number of research studies adopting the phenomenographic method has been growing rapidly in science education (Tight, 2016), hence, we hope this paper can serve as a primer to implement phenomenography in educational practice to improve science learning of students.

## Data Availability

No datasets were generated or analyzed for this study.

## Author Contributions

Both authors made substantial contribution to the conception of the work, drafting the work and revising it critically for important intellectual content, approving the final version of the manuscript to be published, and agreeing to be accountable for all aspects of the work in ensuring that questions related to the accuracy or integrity of any part of the work are appropriately investigated and resolved.

## Conflict of Interest Statement

The authors declare that the research was conducted in the absence of any commercial or financial relationships that could be construed as a potential conflict of interest.
